# Proton pump inhibitors and sensitization of cancer cells to radiation therapy

**DOI:** 10.3389/fonc.2022.937166

**Published:** 2022-08-05

**Authors:** Kassidy A. Hebert, Mark D. Bonnen, Yohannes T. Ghebre

**Affiliations:** ^1^ Department of Radiation Oncology, Baylor College of Medicine, Houston, TX, United States; ^2^ Interdepartmental Graduate Program in Translational Biology and Molecular Medicine, Baylor College of Medicine, Houston, TX, United States; ^3^ Department of Radiation Oncology, University of Texas Health Science Center at San Antonio, Long School of Medicine, San Antonio, TX, United States; ^4^ Department of Medicine, Section on Pulmonary and Critical Care Medicine, Baylor College of Medicine, Houston, TX, United States; ^5^ Dan L. Duncan Comprehensive Cancer Center, Baylor College of Medicine, Houston, TX, United States

**Keywords:** cancer cells, radiation resistance, proton pump inhibitors, radiosensitization, molecular mechanisms

## Abstract

This review article outlines six molecular pathways that confer resistance of cancer cells to ionizing radiation, and describes how proton pump inhibitors (PPIs) may be used to overcome radioresistance induced by alteration of one or more of these signaling pathways. The inflammatory, adaptive, hypoxia, DNA damage repair, cell adhesion, and developmental pathways have all been linked to the resistance of cancer cells to ionizing radiation. Here we describe the molecular link between alteration of these pathways in cancer cells and development of resistance to ionizing radiation, and discuss emerging data on the use of PPIs to favorably modify one or more components of these pathways to sensitize cancer cells to ionizing radiation. Understanding the relationship between altered signaling pathways, radioresistance, and biological activity of PPIs may serve as a basis to repurpose PPIs to restore key biological processes that are involved in cancer progression and to sensitize cancer cells to radiation therapy.

## Background

About 50% of all cancer patients are medically or surgically unfit to have their tumors resected, and require treatment with radiation therapy (1, 2). Ionizing radiation directly or indirectly causes DNA damage to induce cancer cell death. Although an effective treatment strategy, radiation therapy is associated with a number of side effects that may lead to treatment interruption. Additionally, cancer cells can develop resistance to radiation therapy and threaten treatment failure. Previously outlined pathways to radiation resistance include the inflammatory, adaptive, hypoxia, DNA damage repair, cell adhesion and developmental pathway (3). Currently, there are limited number of radiosensitizing agents in development or clinical practice. However, most of these agents invariably target all cells, are very toxic, or largely ineffective. Emerging studies are exploring for strategies to improve the sensitivity and specificity of radiation therapy including searching for safe and effective radiosensitizers among new chemical entities (NCEs) and FDA-approved drug libraries. Intriguingly, proton pump inhibitors (PPIs), FDA-approved for the treatment of gastric reflux, have been evaluated for their anticancer and chemosensitizing effects at preclinical and clinical levels (4–6). Research has demonstrated that PPIs such as pantoprazole, lansoprazole, omeprazole, and esomeprazole possess anticancer and chemosensitizing activity. Recently, studies have indicated that PPIs enhance the effect of ionizing radiation to improve tumor control. This review will explore molecular pathways that are involved in the resistance of cancer cells to ionizing radiation and how PPIs modulate these pathways to overcome radioresistance.

## Inflammatory pathway

Radiation is known to induce the expression of several pro-inflammatory molecules, which can augment pro-survival pathways that ultimately lead to radioresistance. Among the inflammatory molecules that are induced by ionizing radiation is nuclear factor kappa B (NF-κB) (7). NF-κB is a transcription factor for several pro-inflammatory genes and its activation can result in the expression of pro-proliferative and anti-apoptotic pathways to promote proliferation and increase the survival of radioresistant cancer cells (8, 9). Additionally, NF-κB is a known regulator of cyclooxygenase-2 (COX-2) (10) and overexpression of COX-2 has been associated with radioresistance in oral squamous cell carcinoma (11). Radiation is also reported to increase the production of another inflammatory molecule, interleukin-6 (IL-6) (12). IL-6 plays a central role in aggressive tumor growth and treatment resistance (13, 14). In addition, IL-6 induces production of signal transducer and activator of transcription 3 (STAT3), an inflammatory molecule that is known to contribute to radioresistance (15, 16).

For the last 20 years, a number of preclinical and early clinical studies have targeted these inflammatory molecules to induce radiosensitivity (17). In 1999, for example, Pajonk et al. engineered an irreversible binding inhibitor of NF-κB to demonstrate that cancer cells transformed with an NF-kB inhibitor show significantly increased sensitivity to radiation (18). Another study published in the *British Journal of Cancer* also demonstrated an increase in the radiosensitivity of prostate cancer cells treated with the NF-κB inhibitor DHMEQ. The authors found that treatment with DHMEQ reduced the ability of NF-κB to bind to its cognate DNA and resulted in decreased survival of cancer cells treated with radiation (19). Chen et al. (9) compared expression profiles of radiosensitive (HK18) and radioresistant human keratinocytes (HK18-IR), and found that NF-κB was significantly activated in the HK18-IR cells, and was responsible for the radioresistant phenotype in the HK18-IR cell line. Importantly, they were able to overcome the radioresistance in the HK18-IR cells upon expression of a dominant negative mutant that inhibited NF-κB.

Researchers have also tested the possibility of radiosensitizing cancer cells by targeting known regulators of NF-κB including cyclooxygenase 2 (COX-2). COX-2 inhibitors have been shown to induce radiosensitivity either by arresting cells at the G2/M phase of the cell cycle or by inhibiting DNA repair. In this regard, Shin et al. demonstrated that celecoxib, a selective COX-2 inhibitor, amplifies radiation-induced G2/M checkpoint arrest (20). In addition, celecoxib was tested in a phase II clinical trial in combination with oxaliplatin, capecitabine and radiotherapy for the treatment of rectal cancer. The study found that the combination of celecoxib with chemoradiotherapy resulted in high rates of pathologic complete response and surgical downgrading (21).

In addition to the NF-κB pathway, other inflammatory molecules have also been evaluated for radiosensitizing activity. For example, a study conducted by Wu et al. found that inhibition of IL-6 led to the sensitization of prostate cancer cells to radiotherapy (22). More specifically, they found that inhibition of IL-6 with an IL-6 silencing vector was able to increase the cell killing effect of radiation *in vitro*, and delayed tumor growth following radiation therapy in an animal model.

Targeting downstream of IL-6 has also been shown to be an effective strategy to induce radiosensitivity. Pan et al, for example, found that the STAT3 inhibitor, Stattic, was able to induce radiosensitivity in multiple nasopharyngeal carcinoma cell lines (23). The authors concluded that the increase in radiosensitivity was due to Stattic’s action to control cell proliferation and induce apoptosis. An independent study confirmed the efficacy of Stattic in increasing radiosensitivity in hepatocellular carcinoma cell lines in part due to its STAT3 targeting activity to control cell migration and invasion (24).

Notably, targeting each of these key molecules in the inflammatory pathway (IL-6, STAT3, NF-kB, and COX-2) have been reported to increase the sensitivity of cancer cells to ionizing radiation. Intriguingly, PPIs have been shown to target each of these biological molecules *in vitro* and *in vivo* (25–29). A study by Huang et al. observed that treatment of gastric cancer cells with pantoprazole resulted in a dramatic decrease in the levels of IL-6 and a significant reduction in the activation of STAT3 (25). Interestingly, they did not see these results in normal human epithelial cells, suggesting that the effects of pantoprazole were cancer cell-specific. PPIs have also been shown to alter levels of COX-2. In a murine model of colitis-induced colorectal cancer, a study found that mice treated with omeprazole had reduced COX-2 expression (27).

Moreover, several studies have reported that PPIs are able to regulate the expression and activity of NF-κB. Handa et al. found that treatment with omeprazole and lansoprazole significantly decreased the activation of NF-κB in normal human umbilical vein endothelial cells (HUVECs) (26). They also found reduction in NF-κB translocation in HUVECs and in a gastric cancer-derived cell line that had been inflamed with *H.pylori* (26). Finally, Geeviman et al. (28) reported that pantoprazole decreased NF-κB signaling in glioma cells. Compartmentalization study of NF-κB showed that cells treated with pantoprazole only had relatively low levels of NF-κB in the cytosol, but NF-κB in vehicle-treated cells was found in higher levels in both the nucleus and the cytosol. Furthermore, the authors used luciferase assay to demonstrate that the cancer cells had decreased levels of NF-κB regulated genes (e.g. COX-2, iNOS, cyclin D). Ultimately, treatment with pantoprazole was shown to arrest cells in G0/G1 phase of the cell cycle and induce apoptosis. In a study of normal human monocytic (THP-1) cells, lansoprazole was shown to inhibit several pro-inflammatory molecules including NF-κB (29).

Overall, given the promise of inhibiting the inflammatory pathway to overcome radioresistance, and the ability of PPIs to modulate these inflammatory molecules, subsequent work should focus in determining whether PPIs are able to modulate cancer inflammation and enhance the sensitivity of cancer cells to ionizing radiation *in vitro* and *in vivo*.

## Adaptive pathway

Adaptive radioresistance occurs when initial exposure to radiation induces mechanisms of radioresistance. After an initial exposure to radiation, cancer cells can undergo biological changes that allow them to become resistant to subsequent radiation exposure. One of the changes that confers resistance to radiation is the cells’ ability to increase the amount of cyclin D1. Cyclin D1 regulates the transition of cells from the G1 into S phase, and high level of cyclin D1 is correlated with poor prognosis and negative cancer outcomes (30, 31).

Increased levels of cyclin D1 is also observed after exposure to low doses of radiation (32), leading to the speculation that cyclin D may be responsible for the radioresistance observed following initial exposure to ionizing radiation. Ahmed et al. (33) found that when cyclin D was knocked-down using small interference RNA (siRNA), the tested cells (i.e. normal human keratinocytes) were not able to acquire radioresistance even when they were primed with a low dose of radiation. The authors reasoned that an increase in cyclin D1 expression caused radioresistance in part because cytoplasmic cyclin D1 binds to the pro-apoptotic protein and Bcl-2 partner Bax. The interaction between Bax and Bcl-2 is important for the maintenance of mitochondrial membrane potential and mitochondrial apoptosis. By contrast, pretreatment with low dose radiation prevents loss of mitochondrial membrane potential that follows exposure to higher doses of radiation. However, dysregulation of cyclin D1 exacerbates loss of mitochondrial membrane potential even when cells are pretreated with low dose radiation. Mechanistic studies revealed that cyclin D1’s effect in mitochondrial apoptosis, rather than its role in cell cycle, is responsible for its contribution to radioresistance. Recent studies have targeted cyclin D1 as a strategy to overcome radiation resistance in tumors (34).

Importantly, PPIs have been shown to reduce levels of cyclin D1. Assessment of mucosal samples from 60 Barrett’s esophagus patients found significantly fewer alterations in cell cycle proteins in patients placed on PPI therapy than those on alternate antacids such as histamine receptor antagonists (H2RAs). Notably, patients on PPIs showed decreased levels of cyclin D1 (35). Additional studies found that the expression of cyclin D1 is significantly reduced in pancreatic and colorectal cancer cells treated with PPIs (36). Given their FDA-approval, the pleiotropic effect of PPIs to maintain the expression of Bax and to decrease cyclin D1 levels may be an attractive strategy to overcome the resistance of cancer cells to radiation therapy. By decreasing cyclin D1, PPIs are expected to restore the Bax-Bcl2 balance and allow mitochondrial apoptosis upon exposure of cancer cells to ionizing radiation.

In addition to its role in inflammatory pathway, NF-kB is also involved in adaptive pathway. The ability of PPIs to decrease the level of NF-κB may therefore have a pleiotropic effect in sensitizing cancer cells to radiation therapy. Previous work has shown that radiation-induced expression of NF-κB is a significant contributor to radioresistance. For example, a study by Cao et al. (37) demonstrated that the expression of human epidermal growth factor receptor 2 (HER2) in breast cancer cells is induced by radiation, and that NF-kB is necessary for the transcriptional activation of HER2. HER2, a common oncogene in breast cancer, turns on pro-survival signaling networks that are responsible for aggressive and radioresistant cancer phenotype (38).

## Hypoxia pathway

Hypoxia is a significant barrier that confounds the effectiveness of radiation therapy in part because the cytotoxic effect of ionizing radiation depends on the generation of reactive oxygen species (ROS) (39). Hypoxia activates the phosphatidylinositol-3-kinase (PI3K)/AKT pathway, which regulates activation of hypoxia inducible factor 1 α (HIF-1α), a protein important for cell survival in an oxygen-deprived environment. Studies have shown that inhibition of the PI3K/AKT pathway results in reduced expression of HIF-1α and sensitization of hypoxic cells to apoptosis (40). In a study by Burrows et al, it was found that the PI3K pathway was overly active in thyroid carcinomas, and that inhibiting this pathway in an anoxic environment reduced clonogenic survival (41).

Hypoxia also modulates angiogenesis and cell proliferation to influence response to radiation. For example, hypoxia induces HIF-1α, which is a potent stimulator of angiogenesis (42). In addition, PI3K and AKT regulate the expression of vascular endothelial growth factor (VEGF) to control angiogenesis (43). Inhibiting PI3K has been shown to downregulate VEGF (44) and control the angiogenic response stimulated by hypoxia. Pharmacological regulation of this gene network is expected to promote radiosensitization and increase tumor control.

PPIs have been shown to downregulate the PI3K/AKT/HIF-1α pathway (4, 45). This downregulation may oppose hypoxic cells from developing resistance to radiation (46, 47). A recent study found that esomeprazole decreased protein levels of PI3K, AKT, mammalian target of rapamycin (mTOR), and HIF-1α in multiple gastric cancer cell lines (45). This study linked the pharmacological regulation to the control of mTOR through the Tuberous Sclerosis Complex Subunit 1 and 2 (TSC1 and TSC2). In this pathway, TSC1/TSC2 bind with Rheb-GTP to control mTOR Complex 1 (mTORC1) activation. Esomeprazole dose-dependently decreased levels of TSC1/2 and Rheb leading to reduction of mTOR. The downregulation of mTOR led to the suppression of V-ATPase through a negative feedback loop. The authors concluded that the reduction of V-ATPase led to inhibition of the PI3K/AKT/mTOR/HIF-1α signaling pathway and, consequently, to favorable treatment outcomes. These findings are particularly interesting since both PI3K and mTOR inhibitors have been shown to control oxygen tension and reverse hypoxia (41, 46).

## DNA damage repair pathway

The repair of damaged cellular DNA is a physiological process deployed by normal cells to ensure proper cell division including faithful replication of their genetic material. This process also prevents normal cells from accumulating mutations and becoming malignant. However, these pathways are also utilized by cancer cells to increase their fitness and evade immune defense mechanisms. Conversely, several anticancer drugs target DNA damage repair pathways to increase mutation burden in order to control the growth and expansion of cancer cells. At molecular level, when DNA breaks occur, PI3 Kinases initiate the damage repair signaling pathway, leading to the activation of downstream targets including the histone protein γH2AX, checkpoint kinase 2 (CHK2), breast cancer gene 1 (BRCA1) and the tumor suppressor protein p53. When p53 is stabilized by phosphorylation, it can upregulate the expression of p21 and induce G1 cell cycle arrest (48, 49). When CHK2 and BRCA1 are upregulated, they arrest cells in the S and G2/M checkpoints (50). When cancer cells are subjected to radiation, DNA damage is induced through base modification and strand breaks. The strand breaks occur in a single DNA strand or in both strands. The single-strand break repair pathway fixes single-strand breaks, while homologous recombination (HR) and non-homologues end joining (NHEJ) repair double-strand breaks. Targeting these DNA strand-break repair mechanisms may be an effective strategy to enhance the tumoricidal effect of ionizing radiation. A comprehensive review was published on this topic in 2019 (51).

Poly(ADP-ribose) polymerase (PARP) is a protein involved in all three of these pathways of DNA strand-break repair (52–54). Recently, Wang et al. (55) published a study demonstrating that PPIs suppress NHEJ in breast cancer cells by decreasing fatty acid synthase mediated PARP expression. They used a host cell reactivation (HCR) assay to measure the NHEJ and HR events and found that the PPI lansoprazole reduced NHEJ in these cells. They found that treatment of the breast cancer cell line MCF7 with lansoprazole resulted in arrest of the cells in the G1 phase of the cell cycle, while treatment of another breast cancer cell line MDA-MB-468 caused an S phase arrest. The authors further investigated the ability of PPIs to enhance the effect of cancer therapies that rely on DNA damage, and found that they were able to augment the efficacy of both chemotherapy and radiation. Moreover, the study included retrospective analysis of 6754 breast cancer patients separated into groups on the basis of PPI therapy. Encouragingly, it was found that PPIs added to standard of care improved overall survival and reduced recurrence rate compared to standard of care alone. Similarly, a study from our lab demonstrated that the combination of radiation and PPI was more effective in controlling cancer cell growth than treatment with radiation alone. We also found that the combination treatment resulted in more DNA double-strand breaks, as shown by an increase in the DNA double-strand break marker, γH2AX, than in cancer cells treated with radiation alone (56).

## Adhesion pathway

Cancer cells adherent to extracellular matrix (ECM) have a better chance of survival after irradiation than cells that are non-adherent (57). Cell adhesion-mediated radioresistance occurs in many cancer types that affect the colon, cervix, lung, prostate, pancreas, and head and neck (58). Researchers studying this phenomenon found that overexpression of epithelial cell adhesion molecule (EpCAM) in prostate cancer cells is associated with chemoresistance and radioresistance (59). Interestingly, knockdown of EpCAM using siRNA in several prostate cancer cells has been shown to increase sensitivity to both chemotherapy and radiation. An *in vivo* study also showed that knockdown of EpCAM in a prostate cancer cell line prior to engraftment into mouse models led to increased radiosensitivity and prolonged survival of the tumor-bearing animals (60). Additional studies have compared the expression of other adhesion molecules in radioresistant and radiosensitive breast cancer cell lines and found that the resistant cancer cells have increased expression of intercellular adhesion molecule-1 (ICAM-1) and vascular cell adhesion molecule-1 (VCAM-1) (61).

Notably, PPIs have been shown to reduce cancer cell adhesion and downregulate the expression of several adhesion molecules (62, 63). Specifically, treatment of gastric cancer cells with pantoprazole has been shown to inhibit EpCAM to modulate cell proliferation and enhance chemosensitivity (64). Additional studies in primary airway cells also demonstrated the downregulation of chemotherapy-induced ICAM-1 and VCAM-1 levels using esomeprazole (65).

In addition to changes in the expression of EpCAM, ICAM-1 and VCAM-1, abnormal interaction between integrins and the ECM also plays important role in the development of radioresistance. Cell-cell contacts, as well as interaction of cells with growth factors, integrins and ECM is mediated by the focal adhesion kinase (FAK), a non-receptor tyrosine kinase that localizes to focal adhesions to influence cell migration. Eke et al. showed that β1 integrin signaling mediated through FAK was associated with radioresistance in head and neck cancer cell lines (66). Additionally, FAK overexpression has been shown to prevent radiation-induced mitochondria-dependent apoptosis (67). A study demonstrated that this FAK-mediated control of cell function is pH-dependent, and interaction at cell adhesion sites is enhanced in an acidic pH (68). Accordingly, the low pH and enhanced interaction of FAK in cancer cells was found to enhance the migration of cancer cells. PPIs have been shown to buffer the acidic pH of the tumor microenvironment (69) and targeting tumor acidity with PPIs may mitigate the development of FAK-mediated radioresistance.

## Developmental pathway

Lastly, the developmental pathway plays an important role in increasing resistance to radiotherapy. In this regard, the hedgehog signaling, mostly known for its role in cell differentiation during development, has been reported to allow the maintenance of a cancer stem cell population (70, 71). Cancer stem cells have been reported to contribute to the overall resistance of cancer cells to chemotherapy and radiation (72, 73). A study by Gan et al. found that hedgehog signaling contributes to radioresistance in head and neck squamous cell carcinoma cell lines (74) and inhibitors of the pathway have been demonstrated to result in improved tumor control (75–77). Intriguingly, it has been shown that PPIs can inhibit hedgehog signaling (78) and that they may represent a novel strategy to prevent the hedgehog pathway from maintaining cancer stem cells and radioresistance.

In addition, the Wnt/β-catenin and Notch signaling are development-related pathways that are pathologically involved in cancer progression and radioresistance. For example, a study found that Wnt was upregulated in cancer cells that survived radiation therapy in a model of glioblastoma. It was also found that pharmacological inhibition of the Wnt pathway decreased the survival of glioblastoma cells and revealed Wnt as a viable therapeutic target (79). A study of human gastric adenocarcinoma cells demonstrated that inhibition of V-ATPases with the PPI pantoprazole impairs the Wnt/β-catenin signaling pathway resulting in anti-proliferative and anti-invasive effects (80).

Similarly, studies of the Notch pathway have reported that components of this pathway are often overexpressed in cancer. A study that tested the efficacy of a clinically-approved Notch inhibitor found that regulation of this pathway in combination with radiation therapy was able to improve survival outcomes and slow tumor growth in xenograft models *in vivo* (81). Here, it is important to note that just like the Wnt/β-catenin signaling, the V-ATPase pumps have been shown to be required for activation of the Notch signaling (82). Since PPIs are known to inhibit V-ATPases, the Notch pathway presents another opportunity to control tumor progression and radioresistance (83).

## Tumor acidic microenvironment and cancer

Since the PPIs possess anticancer activity against several cancer cell types (4), it is conceivable that a common feature of cancer cells such as the acidic tumor microenvironment are potential PPI targets in addition to or instead of the six pathways discussed above. Because cancer cells are highly proliferative, they use glycolysis as their main source of energy in support of their growth and expansion. As a result, they convert the glucose to lactate in a phenomenon known as the “Warburg effect”. This in turn causes accumulation of protons [H^+^] in intracellular compartments. To control the intracellular and extracellular pH, cancer cells express various proton pumps including the V-ATPases. The sustained acidic tumor microenvironment induces molecular changes in the tumor to enable the cells survive low pH and acquire resistance to chemotherapeutic drugs and radiation. For example, the acidic tumor microenvironment plays a key role in uncontrolled proliferation, metastasis, and chemoresistance (84–86). In addition, the acidic tumor microenvironment adversely affects cytotoxic T cells and limits anti-tumor immunity to promote evasion of the immune system (87). In particular, the V-ATPases, which can be targeted by PPIs (88), are expressed in several cancer cell types and are known to promote resistance to anticancer therapy (89–91). By contrast, targeting V-ATPases reduces extrusion of protons and inhibits tumor growth and metastasis (92). In addition, the buffered tumor microenvironment that follows inhibition of the proton pumps allows the accumulation of chemotherapeutic drugs and sensitizes cancer cells to the anticancer drugs (93). These data sets suggest that proton pumps such as V-ATPases are involved in driving chemoradioresistance, and their inhibition using pharmacological or genetic tools increases the sensitivity of cancer cells to chemoradiotherapy. Paradoxically, however, PPIs are reported to interfere with the efficacy of some chemotherapeutic drugs through drug-drug interaction. Examples of these drugs include capecitabine, methotrexate and irinotecan (94). More recent studies indicate that PPIs may also interfere with immunotherapy through disruption of the gut microbiome and consequent disruption of antitumor immune response to checkpoint inhibitors (95, 96). Overall, the anti-tumor effect of PPIs appears to be associated with the inhibition of V-ATPases and the molecular pathways described above, while the pro-tumor effect of PPIs is likely associated with induction of plasma gastrin levels and disruption of the gut microbiota. Therefore, these opposing effects need to be taken into consideration when combining chemotherapy and/or immunotherapy with radiation in the presence of PPIs. In addition, the role of proton pumps other than V-ATPases in the sensitization of cancer cells to anticancer therapy needs to be cursorily examined. The clinical use of drugs targeted to these pumps should facilitate the evaluation of these drugs as anticancer, chemosensitizers and/or radiosensitizers. For example, new potassium-competitive acid blockers (P-CABs) such as vonoprazan are in clinical use for the treatment of reflux esophagitis and gastroduodenal ulcers. Although it may be too early to assess their anticancer potential through retrospective data analysis at this point, the coming few years are likely to provide sufficient data for large database studies to address questions related to the generalizability of proton pumps for oncologic indications.

## Repurposing PPIs for cancer care

It has been over 30 years since the first PPI was approved by the FDA for the treatment of gastroesophageal reflux disease. Ever since, a number of preclinical and clinical studies have indicated that PPIs possess extra-intestinal activities including anticancer, chemosensitizing and radiosensitizing effects. Preclinically, PPIs have shown substantial anticancer, chemosensitizing and radiosensitizing activities that extend beyond gastric and esophageal cancers. Some of the cancer types that showed promising effect upon the addition of PPIs include pancreatic (97), colorectal (27, 98), ovarian (99), prostate (100), breast (101, 102), lung (56), melanoma (103), lymphoma (104), myeloma (105), osteosarcoma (106) and leukemia (107). This broad anticancer activity of PPIs is likely related to the pleotropic effect of the drug targeting cancer cell growth-, metastasis-, and autophagy- related gene networks (4). Clinically, a number of trials are either completed or underway to test the efficacy of PPIs as anticancer drugs. Currently, there are 84 completed or ongoing trials (www.clinicaltrials.gov) that include the administration of at least one PPI to cancer patients. Some of these studies include combination of high dose PPIs with chemotherapeutic drugs such as docetaxel and cisplatin in metastatic breast cancer; drug-drug interaction studies with molecularly-targeted therapies such as regorafenib and lapatinib; and enhancing neoadjuvant chemotherapy. It remains to be seen which of these studies will be able to meet their primary endpoints to guide future clinical trials and standard of care. The prospective clinical trials and the extraordinary number of PPI use by prescription and over-the-counter provide substantial safety data about the acute and chronic effects of PPIs. In this regard, pharmacoepidemiological studies indicate that chronic use of PPIs is associated with risk of infection, osteoporosis, hypomagnesemia, vitamin B12 deficiency, as well as renal- and hepato- toxicities. Therefore, the repurposing of PPIs for oncologic indications including radiosensitization and chemoradiosensitization is likely going to require careful monitoring of participating cancer patients.

## Conclusion

**Figure 1:**
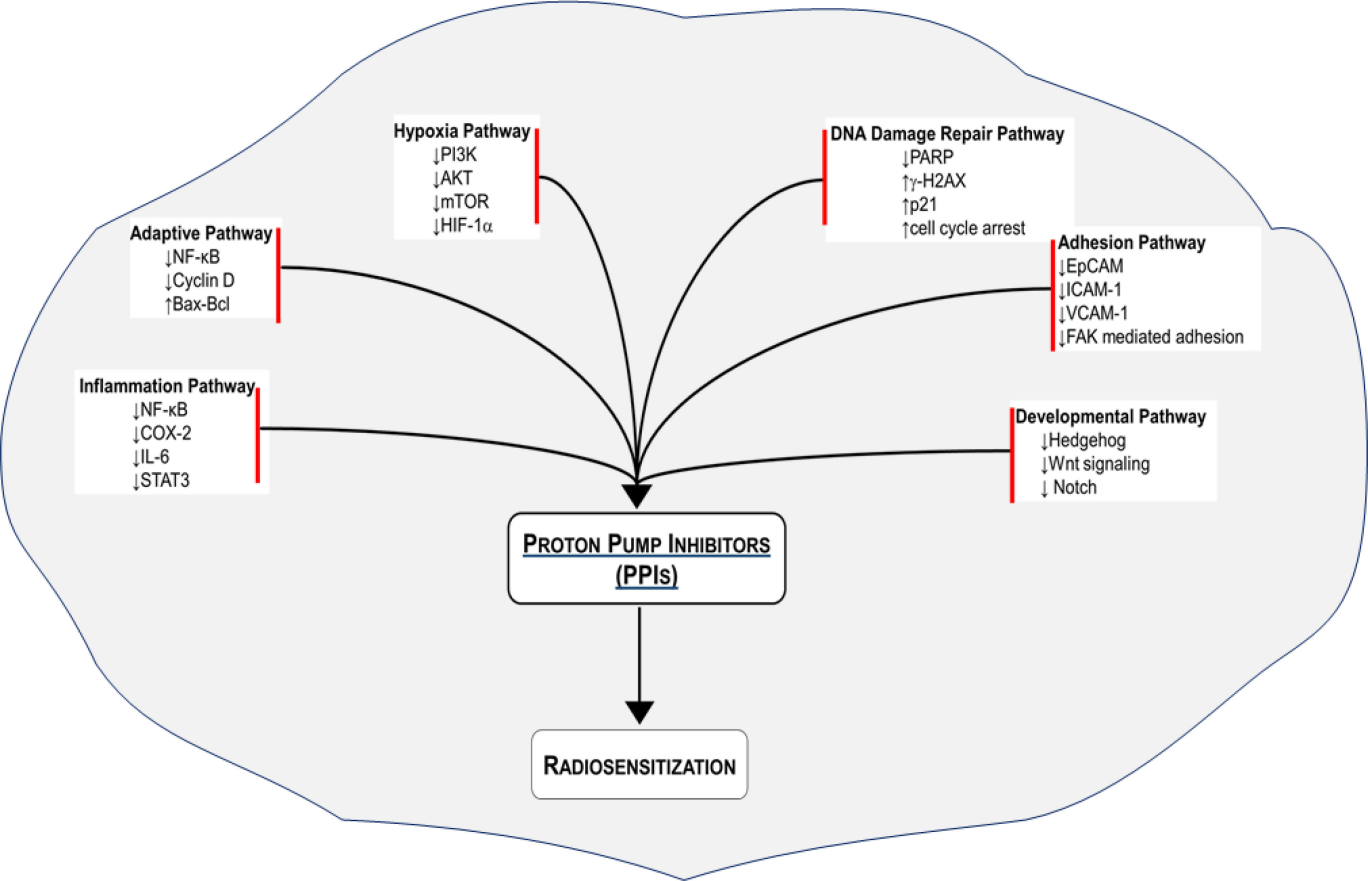
Radiosensitization of cancer cells with proton pump inhibitors (PPIs). Several radioresistance-related signaling pathways are regulated by PPIs to improve the response of various cancer cell types to radiation therapy.

We have outlined several mechanisms by which cancer cells develop resistance to radiation therapy. We have also provided mechanistic insights on how PPIs may overcome the radioresistance (**Figure 1**). A number of studies have demonstrated that PPIs possess anticancer activity including sensitization of cancer cells to chemotherapy. Emerging studies indicate that PPIs may also enhance the effect of ionizing radiation to improve tumor control. We urge that the wide safety margin and pleotropic effect of PPIs should be leveraged to sensitize cancer cells to radiation and chemoradiation therapy. Such an intervention with PPIs is expected to increase the therapeutic index by reducing radiation-induced normal tissue toxicity and improving tumor control (108). Given that PPIs are FDA-approved drugs, they have the potential to be fast-tracked into the clinic. In parallel, mechanistic studies should interrogate the aforementioned and additional molecular pathways to specifically delineate how PPIs sensitize cancer cells to ionizing radiation.

## Author contributions

KH, MB, and YG contributed to conception of the idea discussed in the paper. KH and YG wrote the first draft of the manuscript. KH, MB, and YG wrote sections of the manuscript. MB and YG supervised the manuscript. All authors contributed to the article and approve the submitted version.

## Funding

We would like to acknowledge the Department of Radiation Oncology, and the Department of Medicine at Baylor College of Medicine for overall support. YTG acknowledges funding from the NHLBI (grant numbers K01HL118683; R01HL137703), NIAMS (R56AR077445), the CPRIT (grant number RP190497), and intramural funding from BCM (project ID 2690000104).

## Conflict of interest

YG is an inventor on patents, owned by Stanford University and Baylor College of Medicine, that protect the use of proton pump inhibitors (PPIs) for therapeutic use of new indications. MDB is an inventor on the patent owned by Baylor College of Medicine.

The remaining authors declare that the research was conducted in the absence of any commercial or financial relationships that could be constructed as a potential conflict of interest.

## Publisher’s note

All claims expressed in this article are solely those of the authors and do not necessarily represent those of their affiliated organizations, or those of the publisher, the editors and the reviewers. Any product that may be evaluated in this article, or claim that may be made by its manufacturer, is not guaranteed or endorsed by the publisher.
